# Supplementary material for: The atomistic details of the ice recrystallisation inhibition activity of PVA
Source: Nat Commun. 2021 Feb 26;12:1323. doi: 10.1038/s41467-021-21717-z (PMC7910567; doi:10.1038/s41467-021-21717-z)
Supplement: Supplementary file 1 — Supplementary Information [file 41467_2021_21717_MOESM1_ESM.pdf]

The atomistic details of the Ice Recrystallisation Inhibition activity of PVA

Fabienne Bachtiger,[1] Thomas R. Congdon,[1] Christopher Stubbs,[1] Matthew I. Gibson,[1,2] and Gabriele C. Sosso[1,*]

[1] *Department of Chemistry, University of Warwick, Gibbet Hill Road, Coventry CV4 7AL, United Kingdom*

[2] *Warwick Medical School, University of Warwick, Gibbet Hill Road, Coventry CV4 7AL, United Kingdom*

[*] g.sosso@warwick.ac.uk

We provide supplementary material with further details on:

- Experimental Methods for PVA
- The solvation shell of the methylene (CH_2) groups
- IRI activity and hydrogen bonding data for the restrained simulations, i.e. PVA restrained to compact and linear conformations, where the linear conformations are further restrained to be either parallel or perpendicular w.r.t. the growing ice front.
- Hydrogen bonding data on PVA_{10} and PVA_{20}
- Computational set up for two PVA_{10} polymers along with the corresponding IRI activity
- IRI activity for the block copolymers
- Comparing simulation protocol for finite size effects in ice growth

SUPPLEMENTARY METHODS: EXPERIMENTAL METHODS FOR PVA

A. Materials

All chemicals were used as supplied. Ethyl acetate, hexane, methanol, Petroleum ether 40-60 °C dichloromethane and magnesium sulphate were all purchased from Fisher Scientific at laboratory reagent grade. Deuterated chloroform (99.8 atom % D), dimethyl sulfoxide-d6 (99.9 atom %D), vinyl acetate (97.0%), 4,4'-azobis(4-cyanovaleric acid) ($\geq$ 80.0 %), 2,2'-Azobis(2-methylpropionitrile) (98%), potassium ethyl xanthate (96 %), 2-(methyl bromopropionate) (98 %), hydrazine hydrate solution (50-60 %), and PBS buffer (preformulated tablets) were purchased from Sigma Aldrich. Poly(vinyl amine) Hydrochloride was purchased from polysciences, and was dialysed against MilliQ water using dialysis tubing (MWCO = 1000 Da) and then freeze dried prior to use.

B. Physical and Analytical Methods

^{1}H and ^{13}C NMR spectra were recorded at 400 MHz on a Bruker DPX-400 spectrometer respectively, using deuterated solvents purchased from Sigma Aldrich. Chemical shifts of

protons are reported as δ in parts per million (ppm) and are relative to tetramethylsilane (TMS) at $\delta = 0$ ppm when using DMSO or solvent residual peak (CH_3OH, $\delta = 3.31$ ppm/ DMSO, $\delta = 2.50$ ppm). Size exclusion chromatography (SEC) analysis was performed on an Agilent Infinity II MDS instrument equipped with differential refractive index (DRI), viscometry (VS), dual angle light scatter (LS) and variable wavelength UV detectors. The system was equipped with 2 x PLgel Mixed C columns (300 x 7.5 mm) and a PLgel 5 μm guard column. The eluent is CHCl3 with 2 % TEA (triethylamine). Samples were run at 1mL/min at 30°C. Poly(methylmethacrylate), and polystyrene standards (Agilent EasyVials) were used for calibration. Analyte samples were filtered through a GVHP membrane with 0.22 μm pore size before injection. Respectively, experimental molar mass (Mn, SEC) and dispersity (DJ) values of synthesized polymers were determined by conventional calibration using Agilent GPC/SEC software. The Fourier transform-infrared (FT-IR) analysis was performed on Agilent Cary 630 FTIR spectrometer with diamond ATR system, in the range of 650 to 4000 cm^{-1}. Ice wafers were annealed on a Linkam Biological Cryostage BCS196 with T95-Linkpad system controller equipped with a LNP95-Liquid nitrogen cooling pump, using liquid nitrogen as the coolant (Linkam Scientific Instruments UK, Surrey, U.K.). An Olympus CX41 microscope equipped with a UIS-2 20x0.45∞0-2FN22 lens (Olympus Ltd., Southend on sea, U.K.) and a Canon EOS 500D SLR digital camera were used to obtain all images. Image processing was conducted using Image J, which is freely available from http://imagej.nih.gov/ij/.

C. Synthesis of 2-(ethoxycarbonothioyl)sulfanyl propanoate (EXEP)

Into a round bottom flask was added ethyl acetate (120 mL), potassium ethyl xanthate (4.7 g, 0.02 moles) and then dropwise 2-(methyl bromopropionate) (5.0 mL, 0.03 moles) and the solution left to stir overnight at 60°C. The mixture was filtered to remove insoluble KBr and then concentrated in vacuo. The crude product was partitioned into DCM (100 mL), washed with water (2x100 mL) and sat. brine solution (1x100 mL) and the organic phase was dried using magnesium sulphate (MgSO4). The solution was filtered and concentrated in vacuo, affording the product as a yellow oil. 1H NMR (400 MHz, CDCl3): $\delta = 1.42$ (CH3CH2O, t, 3H), 1.57 (SCH(CH3)CO, d, 3H), 3.75 (COOCH3, s, 3H), 4.40 (SCH(CH3)CO, q, 1H), 4.63 (CH3CH2O, q, 2H). 13C NMR (400 MHz, CDCl3): $\delta = 14$

(CH3CHO), 18 (SCH(CH3)CO), 47 (SCH(CH3)CO) , 55 (COOCH3), 70 (CH3CH2O), 173 (SCH(CH3)COO), 211 (OC(S)S). HRMS (ESI -) m/z: 231.0 [M-H], expected 231.3.

D. Synthesis of Poly(vinyl acetate)

As a representative example, in a glass vial were added vinyl acetate (VA) (3 g, 34 mmol), EXEP (0.07 g, 0.34 mmol) and ACVA (0.09 g, 0.034 mmol). The vial was sealed with a subaseal and the solution thoroughly degassed under a flow of N2 in ice bath for 10 min. The polymerisation mixture was then heated at 70 $\circ$C in oil bath for 8 hours. After an aliquot had been taken for conversion analysis, the sample was diluted in methanol (15 mL) and precipitated into swirling petroleum ether (2 x 200 mL). ^{1}H NMR(CDCl3): δ = 1.72-1.97 (CH2CHOOCH3, br), 1.98-2.15 (CH2CHOOCH3, br), 4.82-5.11 (CH2CHOOCH3, br). Mn(SEC)CHCl3: 2400g.mol^{-1} Mw/Mn(SEC): 1.33. FTIR: C=O 1729 cm^{-1}, C-O 1370 cm^{-1}.

E. Reduction of poly(vinyl acetate) to poly(vinyl alcohol) using hydrazine hydrate solution

To a 50 mL round bottom flask equipped with a stir bar, poly(vinyl acetate) (1.00g) was dissolved in methanol (10 mL) and left to stir until dissolved. Hydrazine hydrate solution (25 mL, 50-60%) was added and the mixture stirred at ambient conditions for 24 h. The reaction was then concentrated in vacuo to remove unreacted hydrazine hydrate and methanol, and the residue diluted with MilliQ grade water (50 mL) and the mixture dialysed using dialysis tubing (MWCO = 1000 Da). The dialysed sample was freeze dried, affording PVA as a white powder. 1H NMR (400 MHz, D2O): δ 4.00 (CH2CHOH, br, 1H), 1.68-1.60 (CH2CHOH, br, 2H) FTIR: O-H (Alcohol) 3278 cm-1, C-O 1418 cm^{-1}.

I. SUPPLEMENTARY NOTE 1: METHYLENE SOLVATION SHELL

To understand what drives PVA to bind to ice, both enthalpic and entropic contributions were investigated (see Fig.7 in main text). To probe the entropic contributions we analysed the change in water phase in the first solvation shell of the methylene groups. This was de-

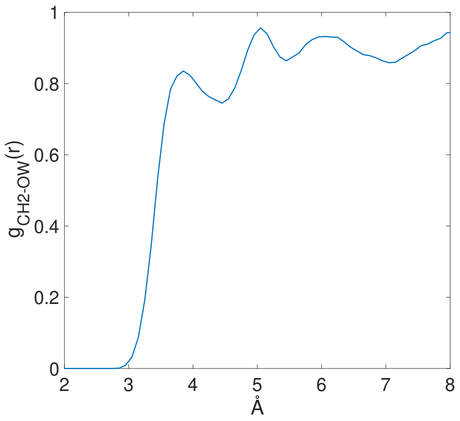

Supplementary Fig. 1: Radial distribution function, $g(r)$, averaged over all methylene groups on PVA_{20} at 300 K. The first minima at 4.5 Å corresponds to the first solvation shell.

termined by examining the radial distribution function, $g(r)$, which for our system describes how the density of the water molecules vary as a function of distance from a reference particle, in this case the methylene groups. Thus, the first minima at 4.5 Å in Supplementary Fig.1 refers to first solvation shell, note that this graph represents an average $g(r)$ taken over the 19 methylene groups on PVA_{20}. Using the $Q6$ output (as discussed in the methodology section) we are then able to distinguish which water molecules within the solvation shell are either classified as liquid (if the methylene group is surrounded by bulk solvent) or ice (if the methylene group is surrounded by the growing ice front) - or a mixture of both.

SUPPLEMENTARY NOTE 2: IRI ACTIVITY AND HYDROGEN BONDING DATA FOR THE RESTRAINED SIMULATIONS

In Supplementary Fig.2 we explore the role of conformation on IRI activity (10 independent simulations for each restrained polymer). Rather than positioning the polymers in such a way which ensured they were all uniformly at a close distance to the ice seed, we took the approach of having the polymers being placed randomly in solution. This ensured that any subsequent binding/IRI activity was more reflective of what might be observed in experiments i.e. we had simulations which had the polymers either placed at the quasi liquid layer or further in the bulk solution or somewhere in between. As a consequence certain polymers might bind to the ice front much later on in the simulation if their initial coordinates were far away from the ice seed. Therefore, to compare the IRI activity for each simulation we

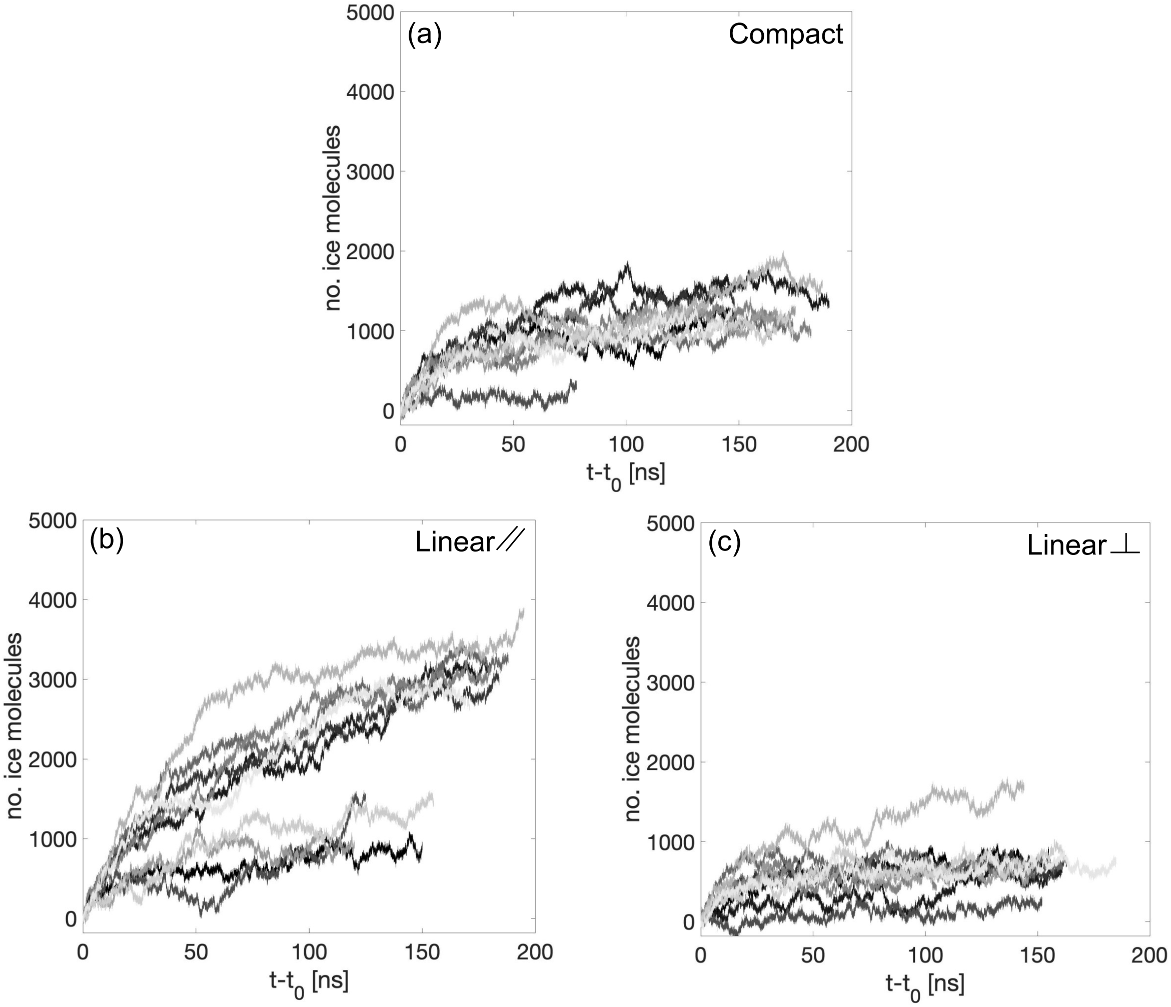

Supplementary Fig. 2: Comparing IRI activity for the restrained polymers. For each simulation t-t_0 represents the time at which 20% of the hydroxyl groups on the polymer chain have bound to ice. From that time onwards IRI activity is monitored in terms of the number of ice molecules found in the growing ice front.

only monitor the number of ice molecules being added to the crystal once the polymer binds to ice, this is determined by a minimum cut-off criteria which is satisfied when at least 20% of the hydroxyl groups bind to ice.

All three conformations are able to exhibit IRI activity, as indicated by a plateauing of the number of ice molecules making up the crystal. However, a linearly restrained polymer positioned parallel w.r.t the growing ice front shows the weakest activity, as indicated by a steady increase in ice molecules for 6/10 simulations. This is due to the polymer becoming engulfed in ice as shown in Fig.3b in the main text and is also supported by the increase in hydrogen bonding between the polymer and ice, as shown in Supplementary Fig.3.

In Supplementary Fig.3 we show firstly, that IRI activity is based on PVA binding to ice which occurs via hydrogen bonding between the hydroxyl (OH) functional groups and ice, secondly, any conformation is able to form hydrogen bonds to ice, and thirdly, once the polymers are bound they do not detach (within the 200 ns simulation time). We also suggest that the % of hydrogen bonds formed as a sole indicator of IRI activity could be misleading; panel b) shows some of the highest % of hydrogen bonding, i.e. those marked with * show up to 80% bonding by the time 200 ns is reached- which is much higher than what is reflected in panel a) in particular, but is also a slight increase from panel c). Despite this, the simulations marked by * are also those which show the polymer progressively becoming engulfed in the ice front with subsequent diminishing IRI activity. Hence we also show that IRI activity is not necessarily proportional to the amount of hydrogen bonds formed by the hydroxyl groups i.e. not proportional to how much of the polymer chain binds ice, which is discussed in section 3 and 4 of the main text.

II. SUPPLEMENTARY NOTE 3: HYDROGEN BONDING DATA FOR UNRESTRAINED PVA_{20} AND PVA_{10}

Supplementary Figs.4 and 5 refer to the hydrogen bonds formed for the 20 independent simulations for PVA_{20} and PVA_{10} respectively. As discussed in the main text in section 2, unrestrained PVA is able to bind to ice in any conformation (typically a random coil) as indicated by the continued existence of hydrogen bonds between the polymers and ice. As mentioned above, we also see here that binding is irreversible on the time scales of the simulation. Furthermore, in Supplementary Fig.4 we see that a random coil conformation, results on average in less than 70% of hydroxyls binding (which is the theoretical value based on a 2:1 binding pattern of a linear polymer restrained perpendicular w.r.t. the ice front), but shows that even fluctuations between 10-70% at any one time is sufficient for a strong enough bind and subsequent IRI activity (see main text). In Supplementary Fig.5 the * refer to those simulations in which the polymers become overgrown in ice, which is also depicted in Fig.4 in the main text.

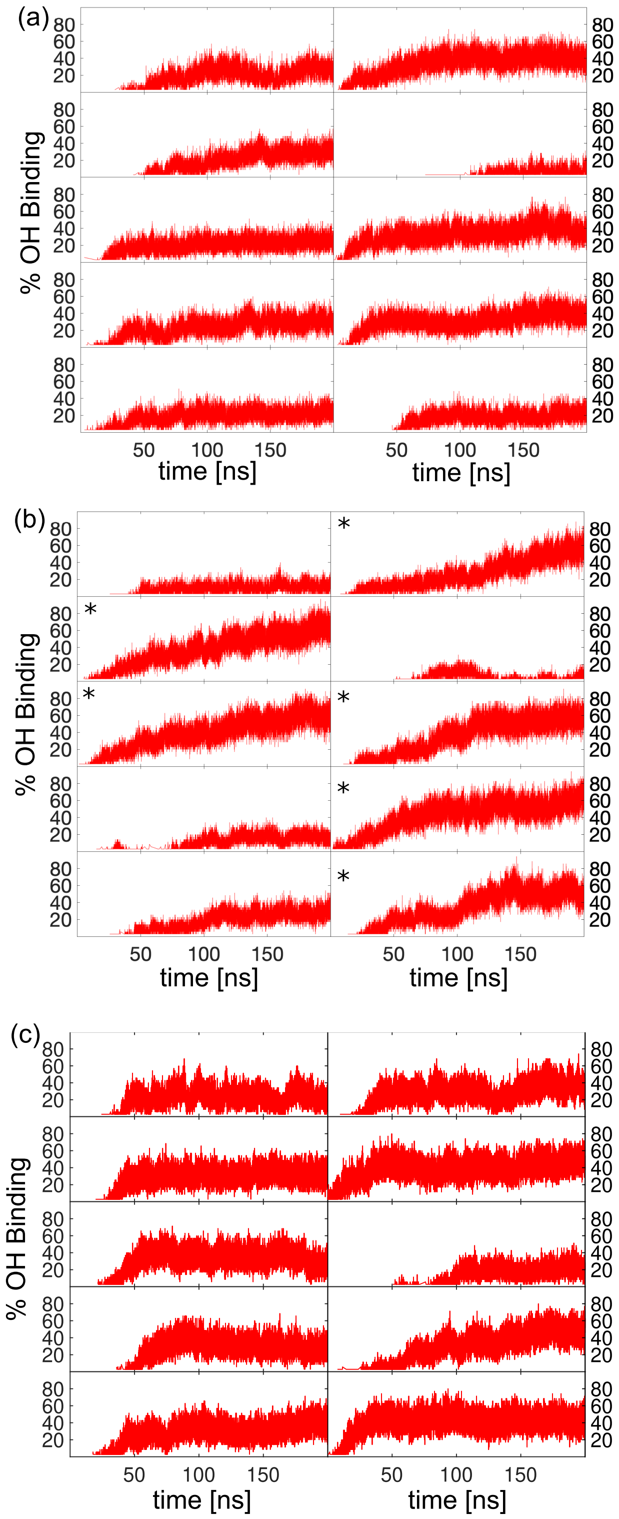

Supplementary Fig. 3: % OH functional groups bound to ice for the restrained PVA_{20} simulations. a) Compact b) Linear parallel c) Linear perpendicular (refer to Fig.1 in main text for depiction). The asterix in b) indicates the polymer has become engulfed and overgrown with ice.

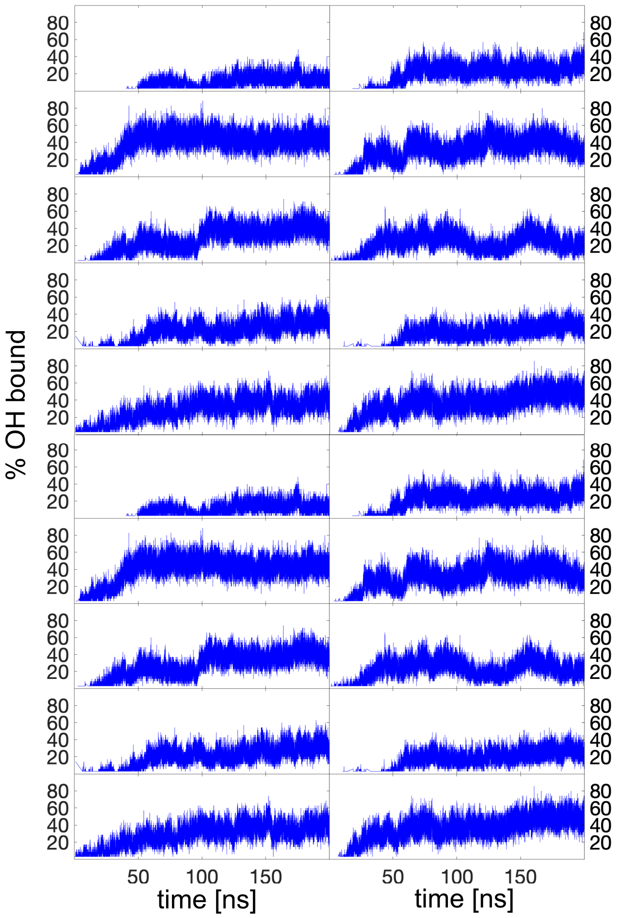

Supplementary Fig. 4: % of OH functional groups hydrogen bonding to ice for unrestrained PVA_{20}. Once bound the polymers do not detach from ice.

III. SUPPLEMENTARY NOTE 4: COMPUTATIONAL SET-UP AND IRI ACTIVITY FOR 2XPVA_{10}

Supplementary Fig.6a illustrates the computational set up for 2 short chain PVA_{10} polymers in the same simulation box, and their corresponding IRI activity (Supplementary Fig.6b). Interestingly, 2xPVA_{10} has the same molecular weight as PVA_{20} yet shows similar activity levels as the simulations with just one PVA_{10} in the simulation box (see Fig.5 in main text). Thus twice the concentration of PVA_{10} is not sufficient for enhanced IRI activity, despite both polymers being able to bind to ice during the simulations. This is further indication of a minimum volume threshold required to be occupied by the polymer once bound to ice.

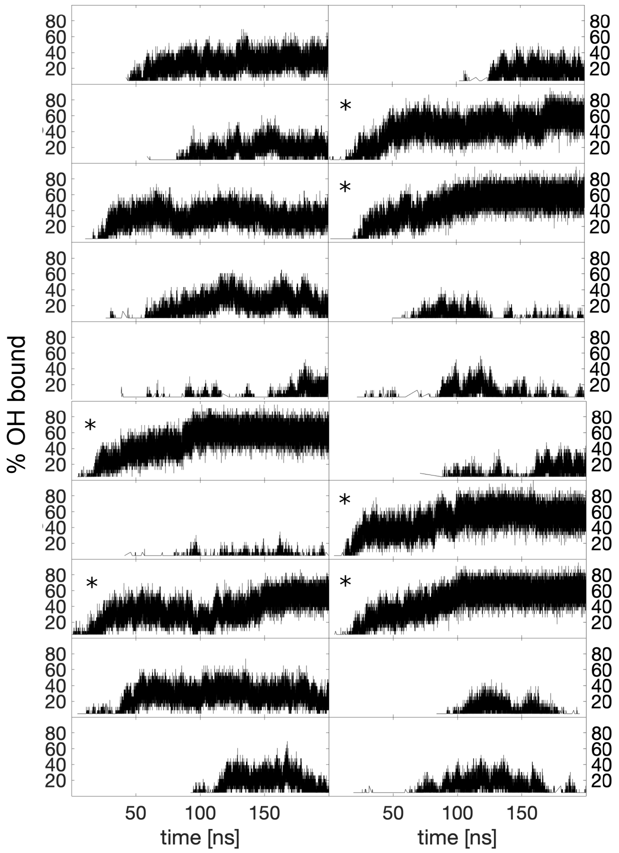

Supplementary Fig. 5: % of OH functional groups hydrogen bonding to ice for PVA_{10}. * denotes simulations where the polymer gets engulfed and overgrown in ice.

IV. SUPPLEMENTARY NOTE 5: IRI ACTIVITY OF THE BLOCK COPOLYMERS

In Supplementary Fig.7a the copolymer with DP = 20 shows the best IRI activity because all 10 simulations show the polymer binding ice -which is likely due to the higher number of hydroxyl groups that are available (13 units) compared to b) and c). For b) and c) the number of hydroxyls is the same (5 units of PVA), but with a DP = 20 and 10 respectively. Here we show that even 5 units is enough for binding and IRI activity and that overall chain length is less important if the functionalisation along the polymer includes an optimum ratio of binding and non-binding groups. For b) and c) some of the simulations showed no IRI activity due to lack of ice binding. Note that the * in b) does not show the polymer becoming overgrown (unlike for the PVA_{10} simulations), but rather uniquely in this simulation initial binding occurred via the amine groups, lasting for around 100 ns followed by the polymer

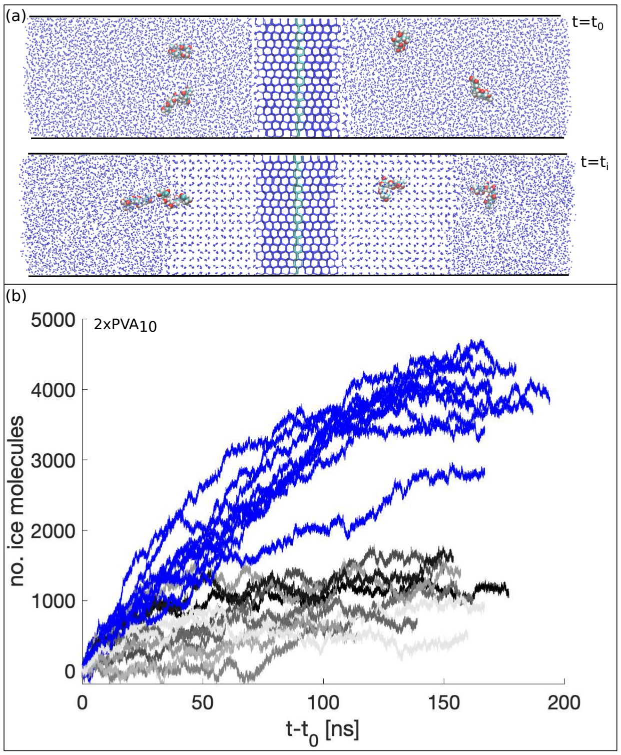

Supplementary Fig. 6: a) Computational set up with 2xPVA_{10}, t_0 provides a snapshot at the beginning of the production run. Note that for the 20 independent simulations PVA can be found anywhere in the water slab, deepening on where it was at the end of equilibration. t=t_i is an example snapshot for one of the blue trajectories found in b) where PVA becomes overgrown. b) corresponding IRI activity, note the simulations in blue indicate where either one or both polymers became overgrown in ice and hence display diminished IRI activity.

dislodging from the ice and moving back into the solution.

V. SUPPLEMENTARY NOTE 6: SIMULATION PROTOCOL TO REDUCE FINITE SIZE EFFECTS IN ICE GROWTH

In all computer simulations there exists a trade-off between simulating a large enough system which reflects reality versus what is practically possible in the confines of resources. We note in the main text (refer to Fig.7) that the control simulations (ice growth only) do not show a linear growth curve (particularly in the latter half of the simulation) -this does not affect our results or mechanistic understanding of the inhibitory properties of the polymers. However, for completeness we examine the finite size effects in closer detail, and suggest our computational set-up to be a good compromise between balancing resources and obtaining physically meaningful statistics.

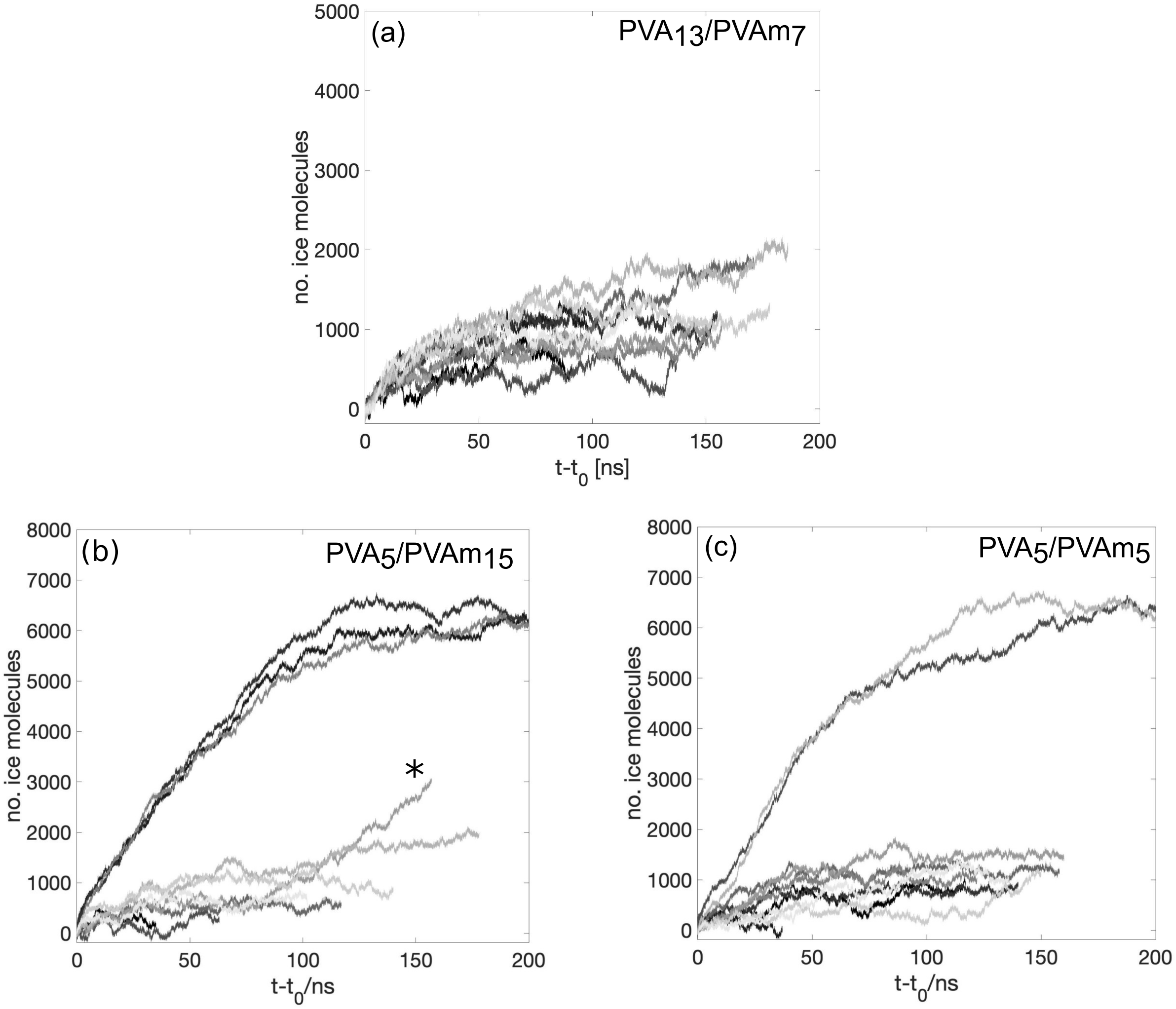

Supplementary Fig. 7: Comparing IRI activity between the three different block copolymers.

Supplementary Fig.8 depicts the growth curve for three different simulation boxes (note that the xy dimensions are kept constant but the water slab increases from 6.5 nm to 11 nm and 22 nm respectively in the z-direction). We see that a water slab of 6.5 nm thickness has virtually no linear growth due to the slowing dynamics of the water molecules at the vacuum/liquid interface and is due to the finite size effect for a system this size. Increasing to 11 nm shows a comparatively better growth rate, which can be improved upon further, if we add a 22 nm water slab to the system. In this case we observe a completely linear growth regime. Due to the available computational resources, studying the IRI activity of PVA in a simulation box with a completely linear growth regime is regrettably unfeasible (167,000 atoms) , and furthermore does not change the underlying observations made in this paper. As such we had to strike a balance and opted for the system with a 11nm water slab.

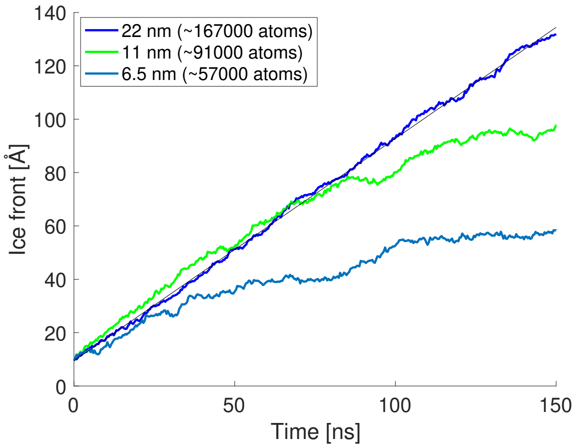

Supplementary Fig. 8: Finite size effects. Different lengths of water slabs (in the z dimension, xy is held fixed) result in different degrees of linear ice growth. The water slab corresponding to 11 nm was the best trade off in terms of computational resources and observing a linear growth regime. As noted in the text, the deviation from linearity does not affect the mechanistic understanding of the inhibitory properties of the polymers investigated in this paper.

Finally, we note that, while the self-diffusion coefficient of TIP4P/Ice water is lower than the experimental value measured at the supercooling of 8 K investigated in this work, the ice growth rate we have computed from our simulations (0.7 Å/ns) is comparable to the experimental value of 0.2 Å/ns reported in Ref. 1. While the growth rate predicted by TIP4P/Ice is indeed faster than the experimental one, we note that the choice of the TIP4P/Ice over e.g. TIP4P/2005 water model (which diffusion coefficient is closer to the experimental value) is motivated by the fact that (a.) TIP4P/Ice provides a much more accurate description of water and ice polymorphs and a much more accurate melting point compared to TIP4P/2005. Indeed, TIP4P/Ice has been consistently used to study ice nucleation and growth in the past [see e.g. Ref. 2] (b.) we are confident that the CHARMM36 - TIP4P/Ice combination of force fields is well suited to perform MD simulations of biomolecules in supercooled water and in contact with ice, including the study of IRI active biomolecules (see Refs. 41, 42, 44–47 in main text.)

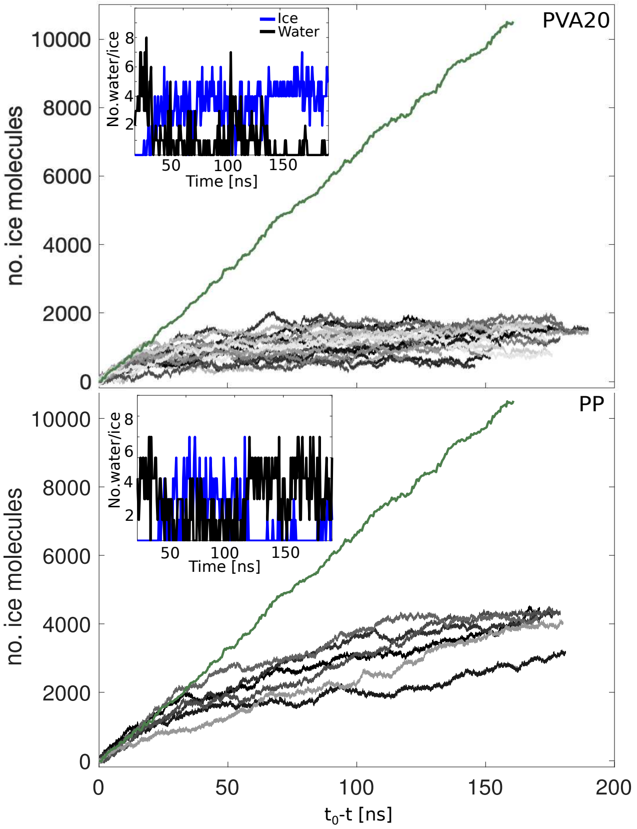

Supplementary Fig. 9: Comparison of the IRI activity of PVA20 and polypropylene (PP), monochromatic lines refer to systems with a polymer, green lines to a system with ice/water only. PP is far less efficient in slowing down ice growth (lower panel) but it does so by means of purely hydrophobic interactions (see inset where we look at desolvation of methylene). Interestingly, the desolvation of the methylene groups of PP is similar to that we observe in the case of PVA (upper panel). PVA20 is much more efficient than PP as an IRI agent as, in addition to the entropic contribution originating from the desolvation of the methylene group, the polymer can further count on the enthalpic contributions due to the hydrogen bonds between hydroxyl groups and ice. (Note that the upper panel and inset are reproduced in the main text as Fig. 5 and Fig. 7. and are reported here for easy comparison).

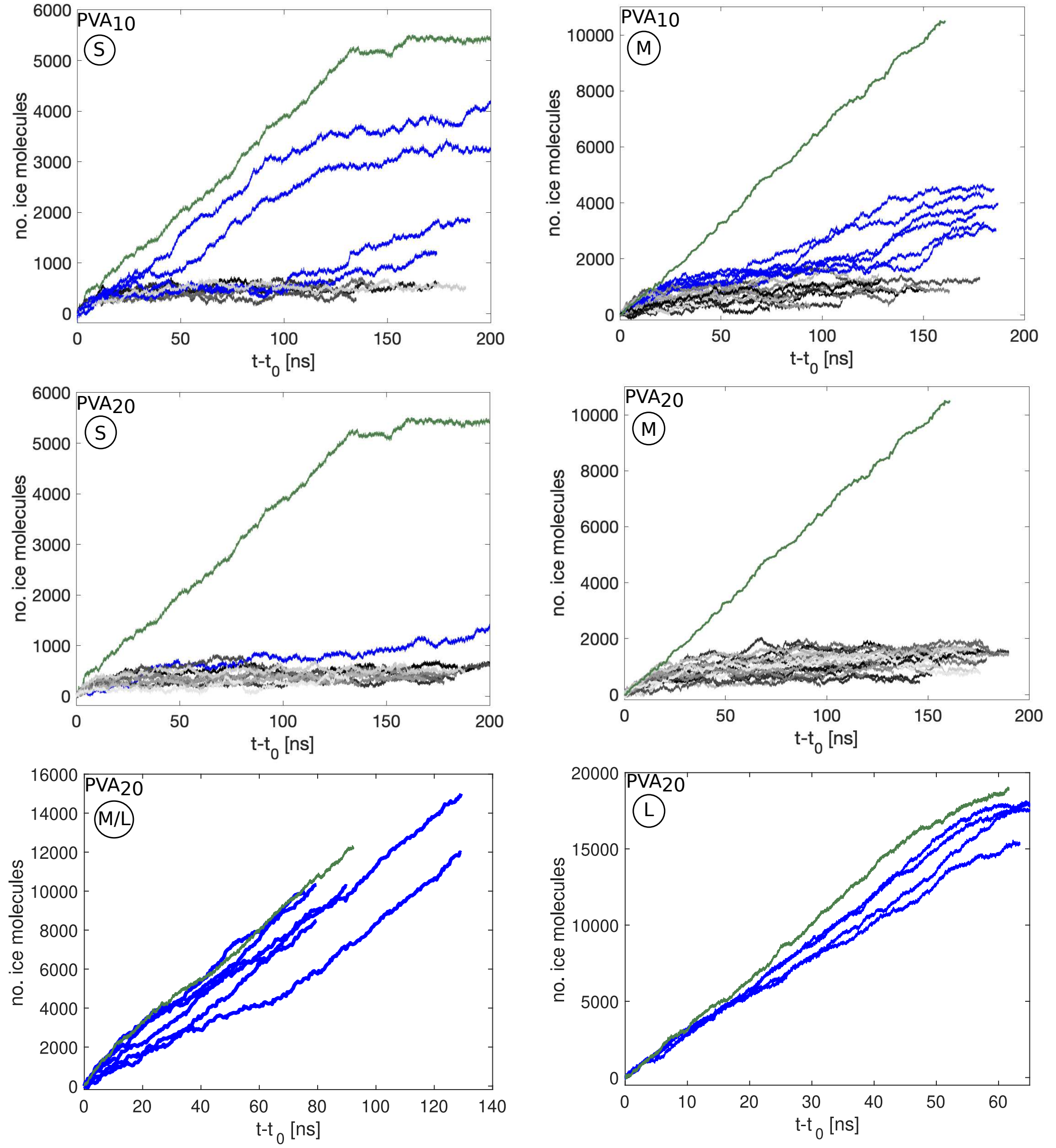

Supplementary Fig. 10: The IRI activity of PVA10 and PVA20 as a function of surface area: 13.3 nm^2 (size S), 26.1 nm^2 (size M, which corresponds to the system size discussed in this paper), 53.4 nm^2 (size M/L) and 124.1 nm^2 (size L). The blue lines correspond to trajectories in which the polymer gets overgrown, while grey lines highlight situations where substantial IRI activity is observed. Green lines correspond to ice growth in the absence of any polymer.

SUPPLEMENTARY REFERENCES

[1] X. Yunato, N. G. Petrik, S. Smith, K. B. D, and G. A. Kimmel, PNAS **113**, 14921 (2016).

[2] H.-A. A and D. P. G, PNAS **112**, 10582 (2015).
